# Pouch Roux-en-Y *vs* No Pouch Roux-en-Y following total gastrectomy: a meta-analysis based on 12 studies

**DOI:** 10.1016/S1674-8301(11)60011-0

**Published:** 2011-03

**Authors:** Liang Zong, Ping Chen, Yinbing Chen, Guohao Shi

**Affiliations:** Department of Gastrointestinal Surgery, Subei People's Hospital of Jiangsu Province, Yangzhou, Jiangsu 225001, China

**Keywords:** gastric cancer, total gastrectomy, reconstruction, meta-analysis

## Abstract

After a total resection of the stomach, the continuity of the gastrointestinal tract can be restored either by Roux-en-Y esophagojejunostomy with or without a pouch. There is still no consensus on the best reconstruction technique. The aim of this report was to derive a more precise estimation of Roux-en-Y esophagojejunostomy with a pouch compared with Roux-en-Y esophagojejunostomy without a pouch. Studies were identified by PubMed and Embase searches, and the inclusion criteria were randomized controlled trials (RCTs) comparing reconstruction techniques between Roux-en-Y with and without a pouch. A total of 12 studies including 1,018 patients were included. The meta-analysis shows that pouch Roux-en-Y does not significantly increase total postoperative complications, anastomotic leakage or mortality. Importantly, there is no significant difference in 5-year survival rates between the two groups. Patients with Roux-en-Y esophagojejunostomy complained significantly less of reflux symptoms and dumping syndrome, and had significantly less severe reflux esophagitis. Quality of life was significantly improved in patients with Roux-en-Y esophagojejunostomy with a pouch compared with patients who received Roux-en-Y reconstruction without a pouch. The results indicate the need for Roux-en-Y esophagojejunostomy with a pouch is a gastric substitute after total gastrectomy by comparison with Roux-en-Y esophagojejunostomy without a pouch.

## INTRODUCTION

Gastric cancer is the second most common malignancy in the world, and surgical resection remains the only curative treatment option. A patient with total gastric resection may undergo various reconstructions[1]–[5]. The method of choice for reconstruction after total gastrectomy for gastric carcinoma still remains controversial. It is well known that, worldwide, the addition of a pouch is only performed in some highly selective large cancer centers. Till now, several studies on this topic have failed to reach a consensus that Roux-en-Y reconstruction with a pouch is a technique of choice. However, Roux-en-Y esophagojejunostomy with a pouch is still considered to be associated with a high incidence of postoperative complications after distal gastrectomy regardless of its main advantage of improving quality of life. In addition, Roux-en-Y esophagojejunostomy with a pouch seems to be highly individual decisions, sometimes by a surgeon's lack of persuasive or objective evidence. The aim of this report was to review all the currently available evidence and derive a more precise estimation of Roux-en-Y esophagojejunostomy with a pouch compared with Roux-en-Y esophagojejunostomy without a pouch by making a systematic analysis. Therefore, data on total postoperative complications, anastomic leakage, mortality, 5-year survival, reflux symptoms, dumping syndrome, reflux esophagitis, eating capacity, serum albumin, quality of life index, operation time, blood loss and hospital stay were extracted to make a meta-analysis on “pouch Roux-en-Y vs. no pouch Roux-en-Y” after total gastrectomy.

## MATERIALS AND METHODS

The entire process of study selection, data analysis, and presentation of results was executed in accordance with the Quality of Reporting Meta-Analysis (QUOROM) statement[6] to ensure the highest quality of this meta-analysis.

### Publication search

Two electronic databases (PubMed and Embase) were searched (last search was updated on 15 October 2010, using the search terms: “total gastrectomy”, “gastric cancer” and “reconstruction”). All eligible studies were retrieved, and their bibliographies were checked for other relevant publications. Review articles and bibliographies of other relevant studies identified were hand-searched to find additional eligible studies. Only published studies with full-text articles were included. When more than one of the same patient population was included in several publications, only the most recent or complete study was used in this meta-analysis.

### Inclusion criteria

The inclusion criteria were as follows: 1) the study addressed the question whether the value of Roux-en-Y esophagojejunostomy with a pouch as a gastric substitute after total gastrectomy was assessed in comparison with Roux-en-Y esophagojejunostomy without a pouch; 2) the study was a clinical trial study; 3) the study contained sufficient data for estimating the odds ratio (*OR*) with 95% confidence interval (*CI*).

### Data extraction

Information was carefully extracted and assessed by two of the authors from all eligible studies. Each of the publications was identified independently. The following data were collected from each study: first author's surname, publication date, study design, patient numbers, length of follow-up, main results and conclusions. Data was included for analysis when at least two randomized control trials (RCTs) analyzed the same specific parameter. We did not define a limit on the minimum number of patients to include a study in our meta-analysis.

### Statistical methods

Comparisons of binary outcome measurements (e.g., complication and the incidence of reflux symptom) among reconstruction techniques were provided by pooled estimates of OR with 95%CI. Effects on quantitative measurements (e.g., length of hospital stay) were analyzed by the weighted mean difference (WMD) approach. Heterogeneity assumption was checked by the χ^2^-based *q* test. *P*-value greater than 0.10 for the *q* test indicates a lack of heterogeneity among studies, so the OR or WMD estimate of each study was calculated by the fixed-effects model (the Mantel-Haenszel method). Otherwise, the random-effects model (the DerSimonian and Laird method) was used[7]. Statistical significance was determined by the *z* test and *P* > 0.05 was considered as statistically significant. Sensitivity analyses were carried out to check if modification of the inclusion criteria of this meta-analysis affected the final results. An estimate of potential publication bias was carried out by the funnel plot. An asymmetric plot suggests a possible publication bias. Funnel plot asymmetry was assessed by the method of Egger's linear regression test, a linear regression approach to measure funnel plot asymmetry on the natural logarithm scale of the OR or WMD. The significance of the intercept was determined by the *t*-test, suggested by Egger (*P* < 0.05 was considered representative of statistically significant publication bias). All the statistical tests were performed with Review Manager Version 4.2 (The Cochrane Collaboration, Oxford, England).

## RESULTS

### Study characteristics

Overall, 30 reports of RCTs comparing Roux-en-Y reconstruction with a pouch and Roux-en-Y reconstructions without a pouch after total gastrectomy for gastric malignancies, published between 1987 and 2010, were found. However, several publications reported on the same trial and the same patient groups differing only in sample size, length of follow-up, or subgroup analysis. Regardless of these partially duplicated reports, a total of 15 independent RCTs could be identified. Three RCTs were excluded, as they did not analyze the parameters for which we sought to analyze (e.g., postoperative complications or the incidence of reflux symptoms or esophagitis or gastritisl), but rather compared cholecystokinin, bacterial counts and total bile acid concentrations over 24 h[8]–[10]. The remaining 12 RCTs, however, of which 2 duplicated RCTs were still included due to the analysis of different and uncorrelated parameters[22],[23], were used to evaluate the value of Roux-en-Y reconstruction with or without a pouch after total gastrectomy. Twelve trials, including 1018 patients, met the inclusion criteria. Of the 12 trials, sample sizes ranged from 20 to 271. The main results of these 12 trials are listed in ***Tables 1*** and ***2***. According to the Cochrane risk of bias tool, all the meta-analyses included only trials with low risk of bias.

**Table 1:** Main characteristics of studies included

RCT	Reconstruction	*n*	Reflux symptoms	Dumping Syndrome	Esophagitis	Postoperative complications	Anastomotic leakage	Mortality	5-year survival
Wei HB[11]	P-RY	63	NA	NA	3	7	NA	1	NA
2008	RY	155	NA	NA	5	12	NA	3	NA
Fein M[12]	J-RY	71	NA	NA	NA	22	8	9	NA
2008	RY	67	NA	NA	NA	21	6	7	NA
Kalmar K[13]	Aboral-RY	22	NA	NA	NA	NA	NA	NA	NA
2001	RY	18	NA	NA	NA	NA	NA	NA	NA
Fuchs KH[14]	J-RY	53	NA	NA	NA	13	5	2	29
1995	RY	14	NA	NA	NA	4	0	1	NA
Hirao M[15]	P-RY	35	NA	NA	NA	5	NA	0	NA
2009	RY	35	NA	NA	NA	5	NA	0	NA
Bozzetti F[16]	P-RY	23	NA	0/15	NA	6	NA	NA	NA
1996	RY	23	NA	1/12	NA	2	NA	NA	NA
Paimela H[17]	J-RY	155	NA	NA	NA	30	7	NA	50-41
2005	RY	116	NA	NA	NA	13	1	NA	34-22
Nozoe T[18]	J-IP	14	1	NA	NA	NA	0	NA	NA
2001	RY	16	3	NA	NA	NA	1	NA	NA
Adachi S[19]	P-RY	10	1	0	NA	2	0	NA	NA
2003	RY	10	6	2	NA	2	0	NA	NA
Kono K[20]	P-RY	23	NA	NA	0	2	0	NA	NA
2003	RY	24	NA	NA	1	0	0	NA	NA
Nakane Y[21]	P-RY	10	3	0	NA	2	0	NA	NA
1995	RY	10	3	1	NA	2	0	NA	NA
Iivonen MK[22],[23]	P-RY	27	11/24	7/24	NA	NA	1/27	NA	15/27
1999-2000	RY	24	9/21	11/21	NA	NA	1/24	NA	14/24

RCT: randomized controlled trial; P-RY: Pouch Roux-en-Y; RY: Roux-en-Y; NA: not available; J-RY: Jejunal Pouch Roux-en-Y; J-IP: Jejunal Interposition Pouch.

**Table 2:** Main Characteristics of studies included (continued)

RCT	Reconstruction	*n*	Operation time (min)	Blood loss (mL)	Hospital stay (d)	Eating capacity in normal size	Albumin(g/L)	Mortality
Wei HB[11]	P-type	63	204±12	NA	NA	NA	38.76 ± 3.41	NA
2008	Orr-type	155	174±6	NA	NA	NA	39.32 ± 3.30	NA
Fein M[12]	J-RY	71	NA	NA	NA	5/17	NA	97.6 ± 23.6
2008	RY	67	NA	NA	NA	5/17	NA	93.6 ± 23.2
Kalmar K[13]	Aboral-RY	22	NA	NA	NA	NA	40.70 ± 3.11	105.9 ± 3.03
2001	RY	18	NA	NA	NA	NA	39.76 ± 3.56	96.5 ± 4.12
Fuchs K-H[14]	J-RY	53	NA	NA	NA	NA	NA	NA
1995	RY	14	NA	NA	NA	NA	NA	NA
Hirao M[15]	P-RY	35	NA	NA	NA	NA	NA	NA
2009	RY	35	NA	NA	NA	NA	NA	NA
Bozzetti F[16]	P-RY	23	NA	NA	NA	NA	NA	NA
1996	RY	23	NA	NA	NA	NA	NA	NA
Paimela H[17]	J-RY	155	171 ± 9	700 ± 100	14 ± 1	NA	NA	NA
2005	RY	116	143 ± 8	500 ± 100	17 ± 2	NA	NA	NA
Nozoe T[18]	J-IP	14	310 ± 91	702 ± 261	NA	NA	NA	NA
2001	RY	16	301 ± 75	568 ± 433	NA	NA	NA	NA
Adachi S[19]	P-RY	10	259±43	678 ± 409	27 ± 6	NA	NA	NA
2003	RY	10	272 ± 60	552 ± 255	33 ± 8	NA	NA	NA
Kono K[20]	P-RY	23	242 ± 28	445 ± 99	NA	NA	NA	NA
2003	RY	24	230 ± 34	499 ± 105	NA	NA	NA	NA
Iivonen MK[22],[23]	P-RY	24	NA	NA	NA	19/24	NA	NA
1999-2000	RY	21	NA	NA	NA	3/21	NA	NA

P-type: P type Roux-en-Y; Orr-type: Orr type Roux-en-Y; J-RY: Jejunal Pouch Roux-en-Y; RY: Roux-en-Y; P-RY: Pouch Roux-en-Y; NA: not available.

### Meta-analysis results

**Fig. 1:**
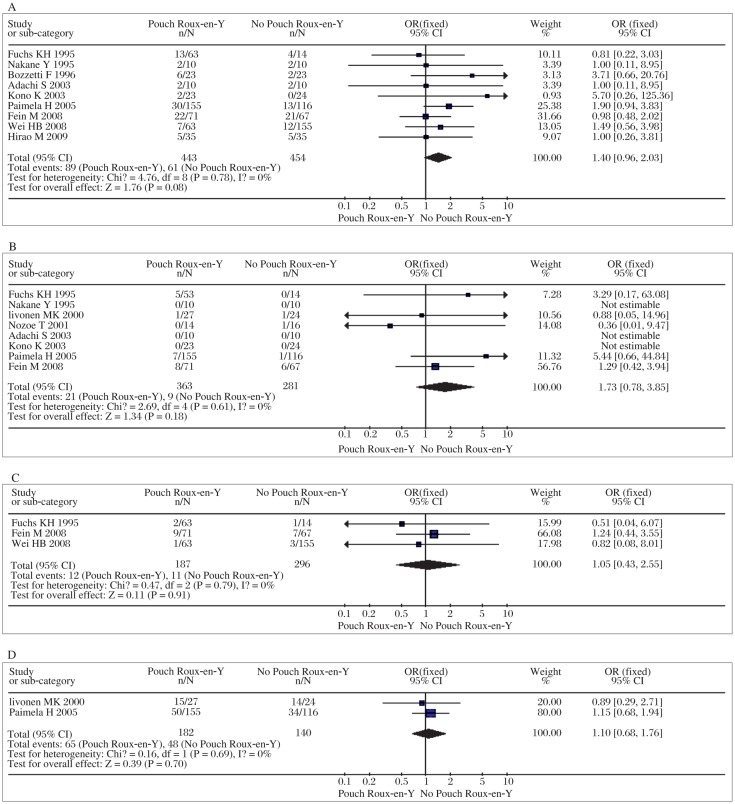
Meta-analyses of parameters of the postoperative course. A: Total postoperative complications. B: Anastomotic leakage. C: Mortality. D: 5-year survival.

**Fig. 2:**
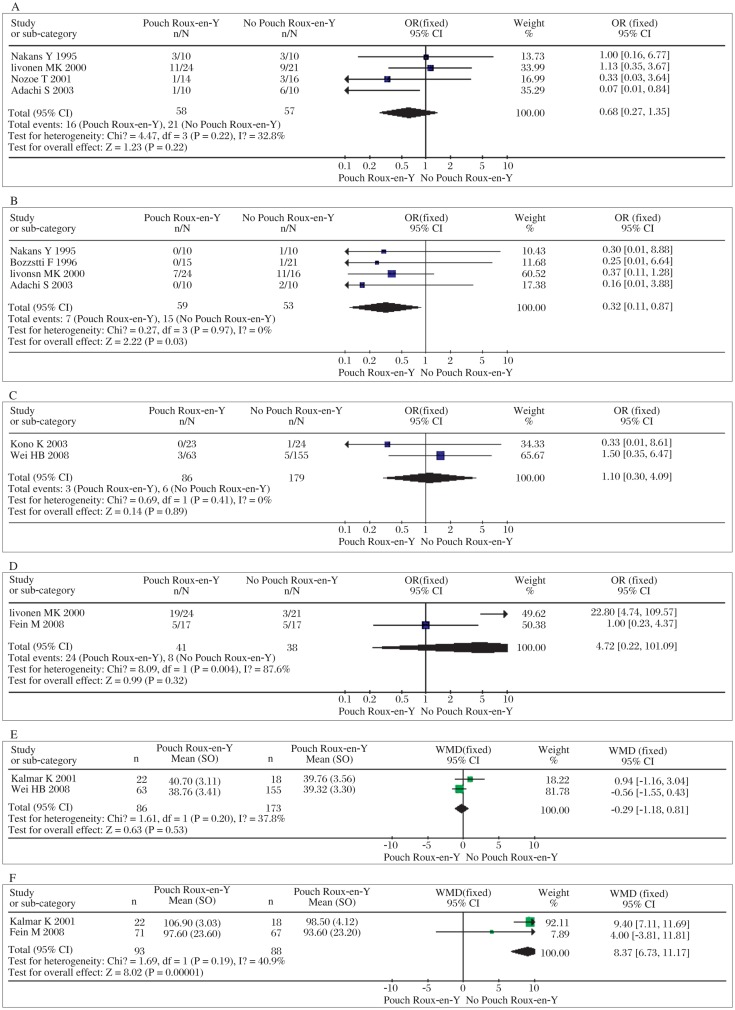
Meta-analyses of parameters of the quality of life. A: Reflux symptoms. B: Dumping syndrome. C: Reflux esophagitis. D: Eating capacity. E: Serum albumin. F: Quality of life index.

**Fig. 3:**
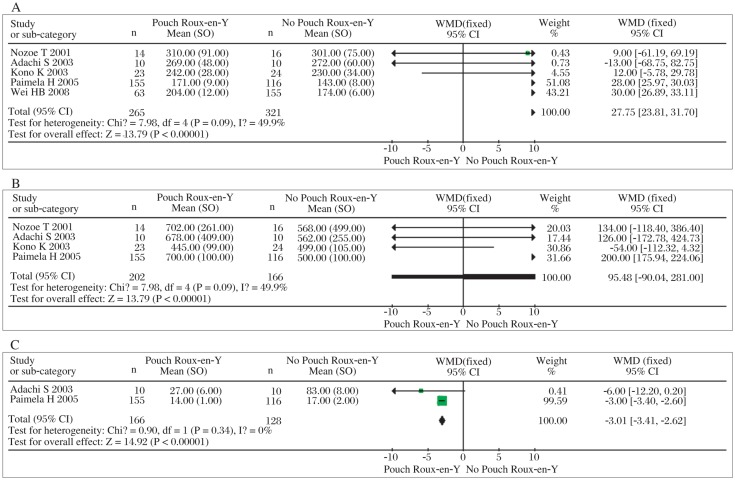
Meta-analyses of parameters of general data of operation. A: Operation time. B: Blood loss. C: Hospital stay.

The results on either total postoperative complications or anastomotic leakage indicate that there was no significant difference between pouch Roux-en-Y reconstruction and simple Roux-en-Y construction (*OR*=1.40, 95% *CI*=0.96-2.03; *P* = 0.08, *P_heterogeneity_* = 0.78 and *OR*=1.73, 95% *CI*=0.78-3.85; *P* = 0.18, *P_heterogeneity_* = 0.61, shown in ***Fig. 1A*** and ***1B***). The meta-analysis on mortality shows that pouch Roux-en-Y reconstruction does not carry significant additional mortality (*OR=*1.05, 95% *CI*=0.43-2.55; *P* = 0.91, *P_heterogeneity_* =0.97, shown in ***Fig.1C***). Similarly, the meta-analysis on 5-year survival showed no significant differences between them (*OR*=1.09, 95% *CI*=0.68-1.77; *P* = 0.72, *P_heterogeneity_* = 0.64, shown in ***Fig. 1D***). Based on the results above, we concluded that pouch Roux-en-Y reconstruction did not relevantly or statistically increase total postoperative complications or anastomosis leakage or mortality compared with no simple Roux-en-Y reconstruction. Moreover, both of them are similar in 5-year survival rate. Meta-analyses carried out for postoperative reflux symptoms, dumping syndrome and reflux esophagitis were performed. Using these parameters, the analyses revealed that pouch Roux-en-Y reconstruction did not reduce the incidence of reflux symptoms or esophagitis, but improved dumping syndrome significantly compared with simple Roux-en-Y reconstruction (*OR*=0.60, 95% *CI*=0.27-1.35; *P* = 0.22, *P_heterogeneity_* = 0.22; *OR*=0.32, 95% *CI*=0.11-0.87; *P* = 0.03, *P_heterogeneity_* = 0.97 and *OR*=1.10, 95% *CI*=0.30-4.09; *P* = 0.89, *P_heterogeneity_* = 0.41, respectively, shown in ***Fig. 2A to 2C***). The patients with a pouch reservoir had a better eating capacity than patients without a pouch reservoir (WMD=4.46, 95 % *CI*=1.73-11.52; *P* = 0.002, *P_heterogeneity_* = 0.004, ***Fig. 2D***). Still, in terms of short-term nutrition, there was no difference in serum albumin content between the two groups (WMD=-0.29, 95 % *CI*=-1.18-0.61; *P* = 0.53, *P_heterogeneity_* = 0.2, ***Fig. 2E***). Meta-analysis showed an improved quality of life in patients who underwent pouch Roux-en-Y reconstruction (WMD=9.54, 95 % *CI*=7.35-11.73; *P* < 0.001, *P_heterogeneity_* = 0.23, ***Fig. 2F***). As for intra-operative data, operation time and blood loss, our meta-analyses report significantly less time but no lower blood loss in simple Roux-en-Y reconstruction than pouch Roux-en-Y reconstruction (WMD=27.75, 95% *CI*=23.81-31.70; *P* < 0.00001, *P_heterogeneity_* = 0.09 and WMD=96.48, 95% *CI*=-90.04-281.00; *p* = 0.31, *P_heterogeneity_* < 0.00001, respectively, ***Fig. 3A*** and ***3B***). Further more, a significantly shorter hospital stay was associated with patients who underwent pouch Roux-en-Y reconstruction opposed to simple Roux-en-Y reconstruction (WMD=-3.01, 95% *CI*=-3.41--2.62; *P* < 0.001, *P_heterogeneity_* = 0.34, ***Fig. 3C***)

### Publication bias

**Fig. 4:**
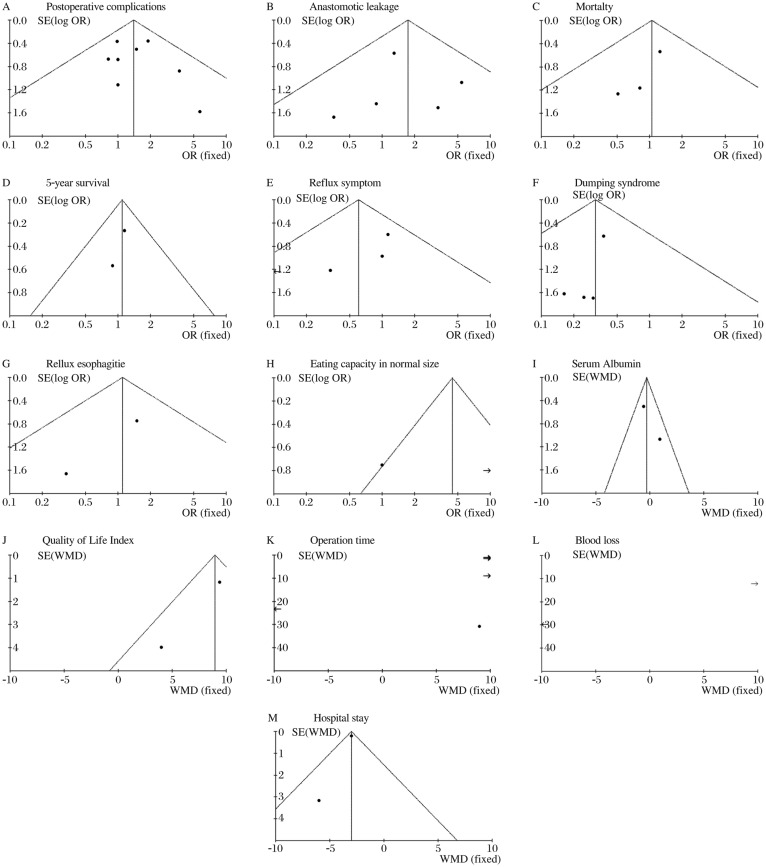
The publieation bias of the retrived literature (Pouch Roux-en-Y *vs* No Pouch) A: Postoperative complications. B: Anastomotic leakage. C: Mortalty. D: 5-year survival. E: Reflux symptom. F: Dumping syndrome. G: Rellux esophagitie. H: Eating capacity in normal size. I: Serum Albumin. J: Quality of Life Index. K: Operation time. L: Blood loss. M: Hospital stay.

Begg's funnel plot was performed to access the publication bias of the retrieved literature (***Fig. 4***).

## DISCUSSION

Surgical treatment plays a predominant role in the management of patients with gastric carcinoma. Total gastrectomy is the most common surgical procedure, which can achieve adequate safety margins in relation to the tumor to offer patients a chance of cure. Various reconstructive procedures can be chosen after total gastrectomy. In Japan, as well as in many other countries, the Roux-en-Y esophagojejunostomy is the preferred reconstruction after total gastrectomy as it is relatively simple to perform and prevents reflux esophagitis. Still, according to some published studies, simple Roux-en-Y construction is not satisfactory with regard to dietary intake, nutrition and quality of life. However, pouch Roux-en-Y reconstruction may provide better dietary intake, nutrition and quality of life in short-term or long-term periods. This alternative has been emphasized as having advantages such as 1) production of the pseudopyloric function to slow the progress of ingested food from a reservoir into the small intestine and reduce the distressing symptoms of the dumping syndrome; 2) provision of a reservoir for digestion and absorption; 3) lessened requirement of frequent meals. Moreover, pouch Roux-en-Y reconstruction may also result in less bile reflux into the esophagus as compared to Roux-en-Y reconstruction. The comparable disadvantages of pouch Roux-en-Y reconstruction include complexity in surgical procedure and the possible development of postoperative complications, especially anastomotic leakage and overall mortality. The potential cause may be additional anastomosis needed to construct. Therefore, Roux-en-Y reconstruction with or without pouch can only be selected by considering the advantages and disadvantages. Supposed that pouch Roux-en-Y reconstruction could be modified to reduce the incidence of postoperative complications and mortality, this procedure would be more strongly recommended for reconstruction after a total gastrectomy. Interestingly, in recent years, with the wide application of the surgical stapler, several studies have found that postoperative complications of pouch Roux-en-Y reconstruction have significantly decreased[24]. Some also argue that this may be the result of improved experience of surgeons. Under this situation, several studies have been designed to provide convincing results to define the optimal reconstruction technique between pouch Roux-en-Y and simple Roux-en-Y for patients necessitating total gastrectomy[8]–[20]. However, the results present no generally accepted consensus, or even absolute opposite opinions. This is partly due to lack of formal meta-analyses and the heterogeneity of reconstruction techniques included in these reviews.

The main objective of surgery for patients suffering from malignant diseases, on the one hand, is to prolong survival time with fewer postoperative complications, and, on the other hand, is to supply a high quality of life in their remaining lifetime. Thus, to make an objective evaluation of surgical procedure, operative parameters such as total postoperative complications, anastomotic leakage, mortality and 5-year survival were collected for comparison in our study. As to postoperative quality of life parameters, reflux symptoms, dumping syndrome, eating capacity, nutrition index and quality of life index were selected from RCTs.

Our meta-analyses indicate no significance in postoperative complications, anastomotic leakage, mortality and 5-year survival between the two procedures. However, pouch Roux-en-Y reconstruction is superior in reducing dumping syndrome and improving food intake. Statistically significant differences are mainly due to the active function of the pouch reservoir. As for reflux symptoms, pouch Roux-en-Y reconstruction is not associated with a lower incidence than simple Roux-en-Y reconstruction because both can play a role in anti-esophageal reflux. The original purpose of Moynihan-type procedure was to reduce the incidence of reflux esophagitis by means of Braun anastomosis. In both simple Roux-en-Y and pouch Roux-en-Y reconstruction, with a 40 to 50 cm distance between the esophagus and the Roux-en-Y anastomosis, the interposed jejunual “Y” limb can prevent esophageal damage from alkaline intestinal secretions. Thus, both the pouch and simple Roux-en-Y reconstruction methods can decrease reflux symptoms and reflux esophagitis.

Finally, serum albumin was measured as a postoperative nutrition parameter. There was no significant difference between pouch Roux-en-Y reconstruction and simple Roux-en-Y reconstruction in the short term postoperatively. That may be explained by total food intake balance. Specifically, when patients with simple Roux-en-Y reconstruction eat less food per meal, they can increase the frequency of daily meals to get an equal total nutrient intake. As a consequence, reduced postoperative complaints and better food intake can extremely improve the patients' quality of life. Furthermore, quality of life index of patients with Roux-en-Y reconstruction reached statistical significance compared with that of patients with simple Roux-en-Y reconstruction during short-term follow-up postoperatively. In our meta-analyses, we also collected and analyzed intra-operative parameters, such as operation time and blood loss, which may correlate with the experience of the surgeon. Because of the wide variation of surgeons' experience and unclear risk of bias, this result showed extreme heterogeneity and a significant difference between the two groups. In other words, pouch Roux-en-Y reconstruction prolongs the operation time without increasing blood loss. Nevertheless, pouch Roux-en-Y reconstruction has been shown to offer patients a reduced length of hospital stay. Possible causes of this finding revealed that the pouch not only reduces the postoperative symptoms, but also contributes to faster postoperative recovery of gastrointestinal function. Moreover, the pouch did not carry additional complications. Overall, each meta-analysis itself, however, also contains several possible shortcomings and bias to be considered[25],[26]. Our meta-analyses prove that Roux-en-Y reconstruction integrated with a pouch after total gastrectomy tends to be a gastric substitute. Finally, we want to emphasize that not only complete oncological resection for cure but also quality of life should be considered in the decision on reconstruction choice.

Owing to the reason of small sample size and potential heterogeneity, it limits us to reach a more precise conclusion. Another meta-analysis on pouch adding reached a similar conclusion to ours[27]. However, a larger number of patients for RCT are required to obtain statistically significant results.
